# The Influence of the COVID-19 Pandemic on the Stress Levels and Occurrence of Stomatoghnatic System Disorders (SSDs) among Physiotherapy Students in Poland

**DOI:** 10.3390/jcm10173872

**Published:** 2021-08-28

**Authors:** Magdalena Gębska, Łukasz Kołodziej, Bartosz Dalewski, Łukasz Pałka, Ewa Sobolewska

**Affiliations:** 1 Department of Rehabilitation Musculoskeletal System, Pomeranian Medical University, 70-204 Szczecin, Poland; mgebska@pum.edu.pl (M.G.); lukas@hot.pl (Ł.K.); 2 Department of Dental Prosthetics, Pomeranian Medical University, 70-204 Szczecin, Poland; bartosz.dalewski@pum.edu.pl (B.D.); rpsobolewski@wp.pl (E.S.); 3 Private Dental Practice, 68-200 Żary, Poland

**Keywords:** COVID-19, stress, stomatognathic system, students

## Abstract

Background: This study is a quantitative analysis examining the impact of the COVID-19 pandemic on the occurrence of stress and stomatognathic system disorders (SSDs) among students of physiotherapy. Objective: To assess stress severity, strategies of coping with stress and the presence of type D personality among physiotherapy students including those with symptoms of stomatognathic system disorders. Material and Methods: The research was conducted from October to December 2020 on a sample of 188 students of physiotherapy. The data were collected using a survey form related to the occurrence of SS disorders symptoms and standardized psychological questionnaires, such as the Perceived Stress Scale (PSS)-10, Mini-Cope, and the type-D Scale (DS14), developed for the purpose of this study. Results: Women experiencing at least one of the SS disorder-related symptoms were characterized by a significantly higher level of stress and a type D personality (*p* < 0.05). Among men, these differences were not statistically significant (*p* > 0.05). On the basis of the strategies of coping with stress, i.e., positive self-reevaluation, discharging and blaming oneself, and taking psychoactive substances, it is possible to predict the intensity of stress during the pandemic in the group of the examined students. Among the reported symptoms of SS, headache was a significant predictor of stress, which was accompanied by an increase in the intensity of stress by nearly 0.2 measurement points. Students with higher levels of stress showed more symptoms of type D personality, and those with more severe symptoms of SS showed higher levels of stress. Conclusions: People prone to stress and having type D personality traits should be assessed for the presence of SS disorders.

## 1. Introduction

The SARS-CoV-2 pandemic has significantly impacted the mental health of populations around the world [1]. As the pandemic-related threat developed, people were forced to change their lifestyle [1]. The economy and its mechanisms, tourism industry, the functioning of culture, and teaching methods at all levels of education have been transformed [1,2]. At medical universities in Poland, necessary recommendations were introduced, i.e., hybrid teaching, limited social and professional contacts, as well as restricted access to university hospitals. As medical college education is largely based on practical skills, students became concerned about their development. Moreover, the uncertainty accompanying senior-year students as to how to fill in the gaps in education could have influenced the level of stress they felt. The pandemic also had a negative impact on attention span and enhanced learning difficulties, which resulted in increased concerns about final exam results.

Social isolation resulting from the pandemic, fear for oneself and loved ones, and financial instability also had a negative impact on the psychophysical health of students. The everyday uncertainty created an environment of anxiety and depression, and the quarantines contributed to a loss of social ties, deepening the feeling of loneliness or anger [3]. These stress factors could lead to the development of health problems associated with the whole body. Psychological factors, especially stress and the ability to deal with it, play a significant role in the etiology of stomatognathic system disorders (SSDs). Coping with lockdown measures was also associated with increased alcohol intake in men [4,5].

Stress that accompanies our daily lives contributes not only to cardiovascular, digestive, and respiratory diseases, but also increases muscle tone of the SS. As a consequence, symptoms such as: bruxism, pain in the temporomandibular joints (TMJs), and headaches may appear. Long-term symptoms of SSDs may contribute to irreversible changes in this system, resulting in impaired chewing, swallowing, speech, and breathing functions [6,7,8,9,10,11,12,13,14,15,16,17,18,19,20]. Additionally, long-term reports about TMJ internal derangements were released [21,22,23,24,25]. Hypothalamus, limbic system and reticular formation are responsible for psychological processes in humans [26]. These centers, through gamma-efferent fibers, which supply the muscle spindles, influence the myogenic activity of the body. In long-term stressful situations, they cause an increase in muscle tonus, also in the facial part of the skull, contributing to the formation of myogenic dysfunctions of the SS [5].

In 1926, Hans Hugon Selye introduced the concept of stress to health sciences. In medical terms, it is defined as a disorder of the body’s homeostasis caused by physical or psychological elements [6,7,8]. It can be caused by mental, physiological, anatomical [7,8], or physical factors [9,10,11,14]. Stressful situations lead to increased muscle tension, which is the body’s natural response to threat. SS muscles, like other skeletal muscles in the human body, can react with increased tension, pain, and/or hypertrophy when strained [7].

People react differently to stressors, which may depend on the personality type of the individual [27]. At the end of the 1990s, the concept of distressed personality (type D) was introduced into the literature, which resulted in an increased interest in the problem of the relationship between personality and somatic diseases [28]. Type D consists of two main dimensions—negative affectivity and social inhibition. Negative emotionality is expressed as a tendency to experience strong negative emotions such as fear, anger, irritation, and hostility. On the other hand, social inhibition is associated with the tendency to refrain from expressing negative emotions and behavior consistent with these emotions. People with type D personalities tend to be worried, tense, and blamed [29]. They are characterized by a pessimistic way of looking at the world, low self-esteem, and low level of satisfaction with life [30]. Type D personality has been found to be a significant predictor of cardiovascular disease, gastric and duodenal ulcer, and skin diseases [31,32,33,34].

Medical college students may be particularly prone to the negative effects of the pandemic. This study is a quantitative analysis examining the impact of the psychological effects of the pandemic on the occurrence of stress and stomatognathic system disorders among students of physiotherapy. The results of this research may help in preparing appropriate future intervention programs and effective prevention strategies in crisis situations at universities [32,35,36,37,38].

The aim of this study was to assess stress severity, coping with stress strategies, and the presence of type D personality in students with symptomatic stomatognathic system disorders. We hypothesized that stress, the ability to cope with it, and the type D personality may influence the development of SS disorders.

## 2. Materials and Methods

The research was conducted from October to December 2020 among randomly selected students of physiotherapy at the Pomeranian Medical University in Szczecin. The sample consisted of 188 participants (125 female and 63 male) aged 20 to 35 years (mean =22.72). All participants agreed to voluntarily participate in the study by signing an informed consent. They were asked to fill in the survey consisting of two parts: the first part included a respondent’s particulars (age, sex, year of study) and ten close-ended questions concerning the symptoms of SSDs and pain (Likert scale) [39,40,41,42,43,44,45]. The second part consisted of standardized psychological questionnaires, PSS10 (perceived stress scale), Mini-Cope (Inventory for measuring coping with stress), DS14 (Type-D scale) [46,47,48,49,50,51,52,53,54]. As a result of the activities performed, 150 participants (study group-P1) with SSD symptoms and pain were selected from the group. The remaining 38 participants who did not report any symptoms from SSD were enrolled into the control group P2.

The sample size was calculated based on the data on a specific population (finite population) and the formula for the minimum sample size was as follows:

$$N_{\min}\;=\;\frac{P(1-P)}{\frac{e^{2}}{Z^{2}}\;+\;\frac{{P(1-P)}^{\prime }}{N}}$$

where:

***P***—estimated fraction size,

***Z***—value resulting from the adopted significance level (α), calculated using the cumulative distribution function of the normal distribution,

***N***—size of the general population (in the case of a finite population),

***e***—maximum estimation error.

After entering the data, namely:

***P***—50%

***Z***—1% (0.01)

***N***—8017

***e***—10%

The finite population sample size equaled: 163

The inclusion and exclusion criteria were as follows:

Inclusion criteria:

- 1st or 2nd year student of physiotherapy learning in a hybrid system (stationary and remote),
- reported symptoms of US disorders (from 6 months),
- age: 20–35,
- a written consent to participate in the study.

Exclusion criteria:

- chronic diseases, including psychosomatic,
- people during or after the treatment of SS disorders,
- pregnancy.

The differentiating factor between the study group (P1) and the control group (P2) was the lack of symptoms of SS disorders in the control group.

Among 1st and 2nd year students of physiotherapy who met the inclusion and exclusion criteria, the study group (P1) and participants without symptoms of SSD (P2) were selected on the basis of the SSD symptom questionnaire.

The main goal of the present study was to compare the groups that are as homogeneous as possible in terms of everyday functioning and exposure to stressful situations while studying during the pandemic. Therefore, a small sample size resulted from the limited number of physiotherapy students.

### 2.1. Characteristics of the Studied Group

Table 1 contains information on the frequency of reported individual symptoms and the average intensity of pain. Descriptive statistics for the entire group and separate statistics for the P1 and P2 subgroups are given. The statistical comparison of these two groups was abandoned due to the lack of occurrence of the studied variables in the P2 group.

As shown in Table 1, the most common symptoms of SS disorders reported by the participants included headaches (68.0%), pain in the neck and shoulder girdle (58.0%), teeth clenching (50.0%) and acoustic symptoms in the temporomandibular joints (33.3%).

### 2.2. Statistical Analysis

The results of the study were statistically analyzed using the IBM SPSS Statistics v. 25 package 9 (IBM, Armonk, NY, USA). To assess the compliance of the empirical distributions obtained in the study with the theoretical normal distribution, typical for general population, the Shapiro–Wilk test for small groups (*n* < 50) and the Kolmogorov–Smirnov test for large groups were implemented. The non-parametric Spearman correlation was used to analyze the relationships between the continuous variables, and the non-parametric Mann–Whitney U test was used to assess the differences between the two independent groups. After standardizing the selected variables, the hierarchical regression model was also used, and the statistical significance index was established at *p* < 0.05.

## 3. Results

Table 2 presents the effect of measuring the frequency of the intensity of stress in the studied groups. The participants scored the PSS-10 scale from low (four raw points, three sten) to very high (35 raw points, 10 sten), and the mean result in the group approaching 19 raw points was within the range of six sten, indicating average severity of stress. The median for the stress level in the P1 group was 20 points and this result was four points higher than in the P2 group—this difference was statistically significant (Z = −2.665; *p* = 0.008). Differences in sten scores were also significant (Z = −2.768; *p* = 0.006). The stress level in the P1 group was higher than in the P2 group. 

Then, the correlation between the intensity of pain and stress in the P1 group (*n* = 150) was analyzed. The measurement data presented in Table 3 indicate that with the increase in the severity of the sense of stress, the severity of headache (rho = 0.359; *p* < 0.001) and pain in the neck and girdle (rho = 0.240; *p* = 0.003) increased as well. 

Table 4 presents the results of measurements of two variables: stress intensity (according to PSS10) and type D personality (according to DS14) among participants who reported one symptom of SS disorders. The analyses were carried out separately for the group of females and males. Males and females differ in this respect; females experiencing at least one SS symptom were characterized by a significantly higher level of stress and a type D personality (*p* < 0.05) compared to P2 group. Among males, these differences were not statistically significant (*p* > 0.05).

Table 5 summarizes the results of a hierarchical regression analysis explaining the intensity of stress experienced by the participants during the COVID-19 pandemic. In each of the five steps of the analysis, the model included successive groups of potential predictors of stress: coping strategies, occurrence of SS symptoms, pain intensity caused by these symptoms, type D personality index, and sociodemographic variables. Before the calculations were made, all data were standardized. It was assessed what percentage of the total variability (variance) in the observed stress intensity can be explained based on changes in individual potential predictors and which of the independent variables are statistically significant predictors. Ultimately, it was possible to explain more than half of the variability in the stress experienced by the participants during the pandemic.

(1) In the first step, the possibility to predict the level of stress severity during the pandemic by applying the strategies of coping with stress among the participants was assessed. Out of 14 strategies, four were significant predictors, which accounted for 33% of the stress variance. 

The analysis showed that the higher the level of positive evaluation, the lower the level of stress. The other three strategies: discharging, taking psychoactive substances, and blaming oneself were positively related to the stress level. 

(2) In the second step, after adding the presence of SS symptoms alone to the model, the explained variance increased to almost 37%, which was a statistically significant change (X2 (10.163) = 2.087; *p* = 0.002). The occurrence of headaches alone became a significant predictor of stress and caused its intensification. All remedial strategies detected in Model 1 as significant maintained their statistical significance (*p* < 0.05).

(3) In the third model, the intensity of the symptoms of the SS was added as a pool of potential predictors. Including them in the model did not significantly increase the percentage of the explained stress variance (X2 (4.159) = 1.468; *p* = 0.214; up to 38% of variance). The severity of pain in the neck and shoulder girdle was a significant predictor—an increase in pain intensity in this area coexisted with a decrease in stress intensity. At the same time, after taking this predictor into account, the strategy of taking psychoactive substances and the occurrence of headaches became statistically insignificant. 

(4) The fourth model included the results of measuring type D personality as a potential stress predictor, which resulted in a significant increase in the explained variance to 55% (X2 (1.158) = 64.591; *p* < 0.001). Of the temporary remedial strategies, only discharging and taking psychoactive substances remained relevant, and both previously diagnosed symptoms of SS lost their relevance. The result on the DS14 scale was the strongest predictor of stress, positively related to the explained variable. The increase in DS14 caused an increase in the level of stress. 

(5) In the fifth model, additionally, a sociodemographic variable was introduced. The gender variable resulted in a significant increase in the explained variance of the stress intensity among students during the pandemic to 58% (X2 (3.155) = 4.109; *p* = 0.008). Among the implemented remedial strategies, the statistical significance was maintained by the discharging and taking psychoactive substances, and among the symptoms of SS, the increase in pain in the neck and shoulder girdle. The sum of type D personality indices was still the strongest predictor, while in the sociodemographic variable—gender—women were characterized by a greater intensity of stress than men.

Table 6 presents the results of the analysis of the correlation between the severity of stress and the styles of coping with it and DS14. It has been shown that the increase in type D personality manifestations was positively and moderately strongly associated with the overall intensity of stress (rho = 0.69; *p* < 0.001); participants with higher levels of stress were characterized by a greater number of type D personality symptoms. Moreover, this personality type was correlated with seven strategies for coping with stress. The severity of symptoms increased moderately with the increase in the tendency to blame oneself in problem situations (rho = 0.51; *p* < 0.001), and it also increased slightly with the increase in the tendency to denial (rho = 0.31; *p* < 0.001), discharging (rho = 0.27; *p* < 0.001), taking psychoactive substances (rho = 0.19; *p* = 0.009) and engaging in other activities (rho = 0.17; *p* = 0.019) and with a decrease in such constructive remedial strategies as positive reevaluation (rho = −0.29; *p* < 0.001) and sense of humor (rho = −0.19; *p* = 0.0010). The overall severity of stress moderately increased with the increase in tendency to blame oneself (rho = 0.46; *p* < 0.001) and weakly with the increase in ceasing activities (rho = 0.32; *p* < 0.001), discharging (rho = 0.31; *p* < 0.001), taking psychoactive substances (rho = 0.27; *p* < 0.001), denial (rho = 0.20; *p* = 0.007), engaging in other activities (rho = 0.15; *p* = 0.042), and with a decrease in the tendency to positively re-evaluate (rho = −0.27; *p* < 0.001), sense of humor (rho = −0.21; *p* = 0.004), active coping (rho = −0.17; *p* = 0.018), planning (rho = −0.16; *p* = 0.028), or seeking emotional support (rho = −0.14; *p* = 0.047).

## 4. Discussion

The outbreak of the COVID-19 pandemic generated a global public health emergency [55,56,57,58]. Taylor et al. reported that psychological responses to previous epidemics and pandemics depended on individual vulnerabilities such as intolerance, insecurity, disease susceptibility, and anxiety [59]. Therefore, the authors of the present study conducted research on the assessment of the level of stress, the method of coping with stress, and the occurrence of symptoms of SS disorders in students of physiotherapy during the COVID-19 pandemic. 

Multiple researchers emphasized the importance of mental stress in shaping the pathology of the SS [17,18,19,20]. Stressors with somatic consequences are largely dependent on the patient’s personality type and the ability to cope with stress. The long-term effects of stressors breaks the body’s adaptive mechanisms and lead to the accumulation of various disorders, including SS disorders [60]. According to the authors’ research on a group of 188 physiotherapy students, the symptoms of SS disorders were found in 150 participants (80%). Research conducted by Emodi-Perlaman et al. showed that the COVID-19 pandemic had a significant adverse effect on the psychoemotional state of Israeli and Polish populations, leading to an increase in bruxism and TMD symptoms [61]. In the author’s own research, symptoms of bruxism, i.e., tooth clenching, were found in 75 participants and teeth grinding in 34 (50% and 22.7%, respectively). In studies by Przytańska et al., patients with high levels of stress reported parafunctional functions, i.e., awake bruxism, almost six times more often [62].

As it results from the conducted research, in the group of students with symptoms of SS disorders, higher scores were observed in the stress severity rating scale compared to the group without SS symptoms. The difference between the groups was statistically significant, which may indicate a significant role of stress in the development of SS disorders. De Medeiros et al. conducted research among Brazilian medical students and indicated that social isolation and stressful situations caused by the pandemic may increase the number of people with TMD symptoms [63].

It is generally accepted that mental stress causes increase in the muscular tension in different parts of the body [64,65]. The body posture adopted in stressful situations related to the so-called “fight or flight” reaction affects muscle contractions within the SS, leading to pain in the head and neck area. Therefore, muscle contractions in the body’s upper quadrant may be a part of a stress-related defensive behavior [14,15,16,17]. In our study, the majority of physiotherapy students reported at least one symptom of SS and pain. The most common symptoms were headache (68%), neck and shoulder pain (58%), and teeth clenching (50%). Many clinical studies seem to confirm the relationship between the occurrence of SS disorders and severe stress, mostly in young people entering adulthood [66,67,68,69]. According to Quintiliani et al., as many as 89.4% of the respondents experienced an increase in perceived stress during the pandemic (66% reported moderate stress and 23.4% high stress) [70].

The authors of the study, by using the PSS 10 scale, found that the average stress intensity among physiotherapy students was in the range of six sten (19 raw points), indicating the average intensity of stress. Similar conclusions were drawn by other authors assessing the intensity of stress in emergency medical students, who also obtained an average value of six [70]. Importantly, in the present study, as many as 46.3% of the respondents obtained results in the range of 7–10 sten, which, according to the current interpretation of the research tool (PSS 10), qualifies them as high stress subjects. It is also worth noting that the maximum score on the PSS 10 scale in the group with symptoms of SSD was higher than in the control group.

The relationship between stress and neurological disorders, and tension headaches and migraines has been expensively described [71,72,73,74]. The results of our own research confirmed the existence of positive correlations between the intensity of stress and the symptoms of SS disorders, because with the increase in the intensity of the sense of stress, the intensity of headache and pain in the neck and shoulder girdle also increased. The above fact should prompt clinicians to evaluate the structures of SS in patients who report the above-mentioned pain symptoms in their history.

According to the literature, SS disorders and higher levels of stress are more common in women than in men [75,76,77,78]. Wang et al. found three times higher levels of depression and health anxiety in women than in men during the COVID-19 pandemic [79]. Moreover, in the research by Liu et al., women were stronger predictors of PTSS symptoms after the pandemic [80]. Our results are in accordance with the abovementioned findings, as we discovered that women with a symptom of SS disorders were characterized by a significantly higher level of stress and a type D personality (*p* < 0.005) compared to men (*p* > 0.05).

The analyses carried out by the authors of the publication with the use of the Mini-COPE test were aimed at examining the activity that students undertake in a stressful situation. These actions are known as “coping methods”. Research conducted by Babicka-Wirkus et al. on a group of 577 students from 17 Polish universities showed that, during the COVID-19 pandemic, students most often used coping strategies such as acceptance, planning, and seeking emotional support [81]. The analysis of the authors’ results did not bring similar conclusions, because out of 14 strategies, four were significant predictors, i.e., positive thinking strategies, discharge, taking psychoactive substances, and blaming oneself. The results also showed that the higher the level of perceived stress, the more frequently the remaining three strategies were used. The level of perceived stress most strongly influenced the frequency of using the discharge strategy, which is a strategy focusing on revealing negative emotions and is associated with a feeling of mental discomfort. The results obtained by the authors allow for the conclusion that maladaptive stress coping strategies in students, especially during the pandemic, may have long-term consequences for their psychophysiological health and academic achievement. According to the research, the presence of SS symptoms did not contribute to changes in the way of coping as all coping strategies retained their statistical significance (*p* < 0.05). 

As it was shown in the study, the intensity of type D personality manifestations was positively and moderately strongly associated with the general intensity of stress (rho = 0.690; *p* < 0.001). Students experiencing higher levels of stress exhibited more symptoms of type D personality, and those with more severe symptoms showed higher levels of stress. In a study by Cho et al. conducted among Korean students, it was observed that type D personality is related to the level of perceived stress. Additionally, it has been concluded that there are gender differences in type D personality, stress, and coping strategies [82]. 

By analyzing the available scientific literature and the results of our own research, it can be determined with high probability that the COVID-19 pandemic increases the intensity of stress and may influence the development of SS disorders.

More research is required to better understand the impact of COVID-19 stressors on stomatognathic disorders.

The major limitation of the present study is the fact that it was carried out in a very specific population. Therefore, the results cannot be extrapolated convincingly to the general population.

## 5. Conclusions

1. The degree of stress intensity influences the occurrence symptoms of the stomatognathic system such as: headache, neck, and shoulder girdle pain.
2. It is important to develop and implement psychological support measures to deal with the COVID-19 pandemic for medical college students.
3. Dental care workers are encouraged to pay more attention to the occurrence of symptoms of SSDs in patients during the pandemic.

## Figures and Tables

**Table 1:** Characteristics of the study group in terms of experienced SS symptoms.

Pain in the Region:	Whole Group, *n* = 188	P1, *n* = 150	P2, *n* = 38
Temporomandibular pain			
Occurrence *	27 (14.4%)	27 (18.0%)	0 (0.0%)
Intensity **	0.32 (0.82)	0.39 (0.90)	-
Headache			
Occurrence	102 (54.3%)	102 (68.0%)	0 (0.0%)
Intensity	1.39 (1.45)	1.73 (1.43)	-
Pain in the neck and pectoral girdle			
Occurrence	87 (46.3%)	87 (58.0%)	0 (0.0%)
Intensity	1.13 (1.35)	1.38 (136)	-
Facial pain			
Occurrence	18 (9.6%)	18 (12.0%)	0 (0.0%)
Intensity	0.20 (0.64)	0.25 (0.71)	-
Acoustic symptoms			
Occurrence	50 (26.6%)	50 (33.3%)	0 (0.0%)
Temporomandibular joint locking			
Occurrence	14 (7.4%)	14 (9.3%)	0 (0.0%)
Teeth clenching			
Occurrence	75 (39.9%)	75 (50.0%)	0 (0.0%)
Teeth grinding			
Occurrence	34 (18.1%)	34 (22.7%)	0 (0.0%)
Muscle tension			
Occurrence	45 (23.9%)	45 (30.0%)	0 (0.0%)

* the number (*n*) and percentage (%) for the occurrence of symptoms ** mean (M) and standard deviation (SD) for pain intensity.

**Table 2:** Basic parameters of the distribution of stress measurement results in the study group (*n* = 188).

	PSS	Mean	SD	Median	Minimum	Maximum	Normality of Distribution
Whole group	Total score	18.82	6.19	18.00	4.00	35.00	0.061
	Sten score	6.27	1.83	6.00	3.00	10.00	<0.001
P1	Total score	19.43	6.18	20.00	4.00	35.00	0.232
	Sten score	6.45	1.83	7.00	3.00	10.00	<0.001
P2	Total score	16.45	5.75	16.00	4.00	29.00	0.751
	Sten score	5.53	1.66	5.00	3.00	9.00	0.036

**Table 3:** Analysis of the correlation between the intensity of stress and the intensity of pain in the P1 group.

PSS	TMD	Headache	Neck and Shoulder Girdle Pain	Facial Pain
Raw score	0.104	0.342 ***	0.241 **	0.139
Sten score	0.090	0.359 ***	0.240 **	0.149

** *p* < 0.01; *** *p* < 0.001.

**Table 4:** Comparison of the mean results of the measurement of stress and type D personality among people suffering from one SS symptom, taking into account gender.

Gender	Compared Variables	Symptoms Occurrence/Group		Group Comparison	
		P1	P2	Z	*p*
Female	PSS	20.63 (5.54)	18.18 (5.45)	−1.922	0.045
	DS14	14.89 (5.25)	11.36 (5.71)	−2.369	0.018
Male	PSS	16.79 (6.73)	12.53 (6.61)	−1.266	0.206
	DS14	14.06 (5.42)	8.75 (4.31)	−1.878	0.060

**Table 5:** Hierarchical regression analysis explaining the intensity of stress among students during the pandemic.

Significant Predictors	Model 1, R^2^ = 0.328		Model 2, R^2^ = 0.368		Model 3, R^2^ = 0.375		Model 4, R^2^ = 0.554		Model 5, R^2^ = 0.579	
	Beta	*p*	Beta	*p*	Beta	*p*	Beta	*p*	Beta	*p*
Positive evaluation	−0.17	0.034	−0.20	0.014	−0.22	0.007	−0.06	0.422	−0.06	0.349
Discharging	0.27	0.004	0.23	0.002	0.24	0.001	0.12	0.027	0.13	0.043
Taking psychoactive substances	0.13	0.041	0.12	0.047	0.11	0.094	0.13	0.027	0.15	0.007
Blaming oneself	0.26	<0.001	0.22	0.002	0.24	0.001	0.04	0.566	0.03	0.686
Headache	-	-	0.20	0.006	0.05	0.700	0.07	0.522	0.05	0.660
Intensified Neck pain	-	-	-	-	−0.31	0.022	−0.20	0.085	−0.23	0.040
DS14	-	-	-	-	-	-	0.56	<0.001	0.53	<0.001
Gender	-	-	-	-	-	-	-	-	−0.20	0.001

**Table 6:** Spearman’s correlation matrix.

Variables	1	2	3	4	5	6	7	8	9	10	11	12	13	14	15
1. DS14	-	-	-	-	-	-	-	-	-	-	-	-	-	-	-
2. PSS 10	0.69 ***	-	-	-	-	-	-	-	-	-	-	-	-	-	-
3. Active coping	−0.14	−0.17 *	-	-	-	-	-	-	-	-	-	-	-	-	-
4. Planning	−0.07	−0.16 *	0.51 ***	-	-	-	-	-	-	-	-	-	-	-	-
5. Positive evaluation	−0.29 ***	−0.27 ***	0.19 *	0.40 ***	-	-	-	-	-	-	-	-	-	-	-
6. Acceptance	−0.03	−0.13	0.21 **	0.29 ***	0.35 ***	-	-	-	-	-	-	-	-	-	-
7. Sense of humor	−0.19 *	−0.21 **	0.01	0.02	0.39 ***	0.40 ***	-	-	-	-	-	-	-	-	-
8. Turning to religion	−0.03	−0.02	0.07	0.15 *	0.18 *	0.12	0.07	-	-	-	-	-	-	-	-
9. Seeking emotional support	−0.12	−0.14 *	0.20 **	0.34 ***	0.35 ***	0.16 *	−0.02	0.11	-	-	-	-	-	-	-
10. Seeking instrumental support	−0.04	−0.04	0.18 *	0.27 ***	0.34 ***	0.19 *	−0.03	0.21 **	0.77 ***	-	-	-	-	-	-
11. Engaging in other activities	0.17 *	0.15 *	−0.01	0.11	0.11	0.15 *	0.15 *	0.03	0.16 *	0.17 *	-	-	-	-	-
12. Denial	0.14 *	0.20 **	−0.06	−0.06	0.05	−0.05	0.12	0.09	−0.17 *	−0.11	0.12	-	-	-	-
13. Discharging	0.26 ***	0.31 ***	−0.07	0.08	0.20 **	0.08	0.01	0.07	0.20 **	0.35 ***	0.26 ***	0.27 ***	-	-	-
14. Taking psychoactive substance	0.19 **	0.27 ***	−0.07	−0.02	−0.08	0.03	0.12	0.03	−0.12	−0.12	0.19 **	0.14	0.12	-	-
15. Ceasing activities	0.31 ***	0.32 ***	−0.45 ***	−0.29 ***	−0.19 **	−0.01	−0.01	−0.03	−0.24 **	−0.09	−0.06	0.27 ***	0.21 **	0.13	-
16. Blaming oneself	0.51 ***	0.46 ***	0.01	−0.09	−0.19 **	−0.10	−0.16 *	0.05	0.14 *	0.01	0.06	0.28 ***	0.25 ***	0.27 ***	0.26 ***

* *p* < 0.05; ** *p* < 0.01; *** *p* < 0.001.

## Data Availability

The data presented in this study are available on request from the corresponding author. The data are not publicly available due to sensitive information.
